# Splenectomy reduces shear stress and inflammation in liver endothelial cells during regeneration after partial hepatectomy in mice

**DOI:** 10.1038/s41598-025-32446-4

**Published:** 2025-12-13

**Authors:** Andrey Elchaninov, Elena Gantsova, Polina Vishnyakova, Maria Kuznetsova, Dmitry Trofimov, Timur Fatkhudinov, Gennady Sukhikh

**Affiliations:** 1 Laboratory of Growth and Development, Avtsyn Research Institute of Human Morphology of FSBI “Petrovsky National Research Centre of Surgery”, 3 Tsuruppa Street, Moscow, 117418 Russia; 2 Laboratory of Regenerative Medicine, Institute of Translational Medicine, Gynecology and Perinatology Named After Academician V.I., National Medical Research Centre for Obstetrics, Kulakov of Ministry of Healthcare of Russian Federation, Moscow, Russia; 3 Research Institute of Molecular and Cellular Medicine, Peoples’ Friendship, University of Russia, RUDN University), Moscow, Russia; 4 Laboratory of Molecular Research Methods, Institute of Reproductive Genetics, National Medical Research Centre for Obstetrics, Gynecology and Perinatology Named After Academician V.I., Kulakov of Ministry of Healthcare of Russian Federation, Moscow, Russia

**Keywords:** Spleen, Liver, Regeneration, Endothelial cells, Cell biology, Diseases, Gastroenterology, Immunology, Medical research

## Abstract

Clinical and experimental findings indicate that splenectomy stimulates liver regeneration. The mechanisms of this effect are uncertain. This study aimed to assess the effect of splenectomy on the state of sinusoidal endothelial cells during liver regeneration after 70% resection in mouse model. Two series of experiments were performed. In the first series, splenectomy was performed on sexually mature male C57BL/6 mice, followed by 70% liver resection 7 days later. In the second series, only 70% liver resection was performed. The animals were withdrawn from the experiment 1, 3, or 7 days post-resection. The measurements encompassed the liver mass recovery dynamics; the AST, ALT, and albumin levels; and the proliferation activity of hepatocytes, as well as gene expression profiles, apoptosis rates, and proliferation activity of liver sinusoidal endothelial cells. Animals that underwent splenectomy before liver resection showed a decrease in the number of endothelial cells positive for VCAM-1 on days 1 and 7 post-resection and VE-cadherin-positive (CD144 +) cells on day 7. Liver resection caused an increase in the relative counts of dying (Annexin-PI +) and Ki67 + endothelial cells in the remnant. The apoptotic endothelial cell counts were significantly higher in the group with preserved spleen on day 1 post-resection, while the number of Ki67 + endotheliocytes was higher in splenectomized animals on day 1 and day 3 post-resection. Splenectomy exerts significant effect on the state of endothelial cells of the sinusoidal capillaries of the regenerating liver. The gene expression profiling studied by microarray technology using Clariom™ S Assay (mouse) revealed specific enrichment of the following signaling pathways in the endothelial cells of regenerating liver in splenectomized animals: PI3K-Akt-mTOR signaling pathway, Focal adhesion pathway, Chemokine signaling pathway. In this case, most of the differentially expressed genes were repressed. The decreased proinflammatory activation of sinusoidal endothelium results in decreased immigration of lymphocytes, notably CD3 + и NK1.1 lymphocytes known to inhibit the regeneration. The results indicate that prior splenectomy mitigates the manifestation of shear stress in endothelial cells of the liver and reduces their pro-inflammatory activation, resulting in lower rates of migration of regeneration-inhibiting lymphocytes to the regenerating liver.

**Supplementary Information:**

The online version contains supplementary material available at 10.1038/s41598-025-32446-4.

## Introduction

Mammalian liver has a unique capacity for regeneration^1^. The most common experimental setting used to study this phenomenon is the liver mass restoration after 70% resection in mice and rats^2,3^. The prevalence of this model is explained by the ease of its reproduction in laboratory rodents. In this model, the regeneration process is accomplished mainly through proliferation of hepatocytes and other cell types in the lobes remaining after resection. The liver mass restoration is completed in 7–10 days after surgery^4^.

It should be noted that, despite the long history of liver regeneration studies in laboratory animals, there is no consensus on which mechanism is responsible for initiating the restoration of the resected mass of the liver^5^. Two main mechanisms are assumed. According to the first hypothesis, the resection boosts endotoxin levels in the blood of experimental animals through the deficiency of endotoxin metabolization by the liver. The increase in endotoxin levels promotes activation of resident liver macrophages which start to produce Il6 and TNFα thereby enhancing the sensitivity of hepatocytes to mitogens, notably HGF released from extracellular matrix. Under the mitogenic stimuli, hepatocytes enter cell cycle and undergo 1–2 rounds of proliferation, after which the liver mass restoration is complete^6^.

According to the second hypothesis, liver resection leads to an immediate change in hemodynamic conditions. Signs of portal hypertension are observed in the vessels of the resected liver, which leads to the development of the so-called shear stress in endothelial cells, primarily in the sinusoidal capillaries. These changes lead, among other things, to the synthesis of various mitogens (HGF, TGFα, etc.) by the endothelial cells themselves, which stimulates the entry of hepatocytes into the mitotic cycle. Restoration of the liver parenchyma and its vascular bed ultimately resolves the portal hypertension^5,7^. The two mechanisms can be assumed to act simultaneously, which makes the liver regeneration process less prone to dysregulation.

Noteworthy, the signs of portal hypertension in the liver are observed not only after its resection, but also in liver fibrosis, cirrhosis and the post-transplantation small remnant syndrome^8,9^. In human pathologies, by contrast with experimental settings, portal hypertension tends to persist and may ultimately lead to depletion of the regenerative potential of the liver^8^. In this regard, various procedures designed to reduce portal hypertension may have a beneficial effect on the liver. One such manipulation is surgical removal of the spleen^10^.

Of all organs and structures of the immune system, the spleen is most closely connected with the liver. The connection widely termed ‘hepatosplenic axis’ is both anatomical, defined by the presence of a portal vein carrying cytokines and other regulatory molecules, and possibly also migratory cells, from the spleen to the liver, and functional, as both organs participate in immune defense, barrier function, and blood storage^11^.

Pathophysiological indications of hepatosplenic axis were initially observed in patients with liver cirrhosis and subsequently studied in animal models. Although liver diseases are often accompanied by pathological changes in the spleen including hypersplenism and splenomegaly^12^, the net influence of the spleen on liver regeneration may vary and the data in this regard are highly controversial^13^.

The literature describes a beneficial effect of splenectomy on the liver mostly post-transplantation or in liver fibrosis. The effect was observed both experimentally and clinically^14,15^. In addition to the apparent link with portal hypertension, other mechanisms for the beneficial effect of splenectomy on the liver have been proposed^16^. At the same time, the effect of hepatectomy on the endothelium of liver sinusoidal capillaries is still understood rather poorly^17^ while being of critical importance in the prospect of new methods for stimulating liver regeneration where the liver sinusoidal endothelial cells (LSECs) are considered as a possible therapeutic target.

This study explores the effect of splenectomy on the state of endothelial cells of the liver sinusoidal capillaries after 70% experimental resection.

## Methods

### Animals

The study used C57BL/6 male mice, body weight 20–22 g, received from the “Stolbovaya” facilities (Moscow region, Russia).

The animals were housed in plastic cages at 22 ± 1 °C on a 12-h light/12-h dark cycle with light on from 6:00 am to 6:00 pm, with free access to standard food for laboratory rodents and water. The conditions for keeping the laboratory animals complied with "The International Guiding Principles for Biomedical Research Involving Animals" of 1985, the rules for laboratory activities in the Russian Federation (Order of the Ministry of Health of the Russian Federation of 91.06.2003 No. 267) and the law "On the Protection of Animals from Cruelty" Chapter V of Article 10, 4679-GD of 01.12.1999. The study was approved by the Ethics Committee of the A.P. Avtsyn Research Institute of Human Morphology (protocol No. 29 (5) dated November 8, 2021). This study is reported in accordance with the ARRIVE guidelines (PLoS Biol 8(6), e1000412, 2010) for animal research.

### Model

**Fig. 1:**
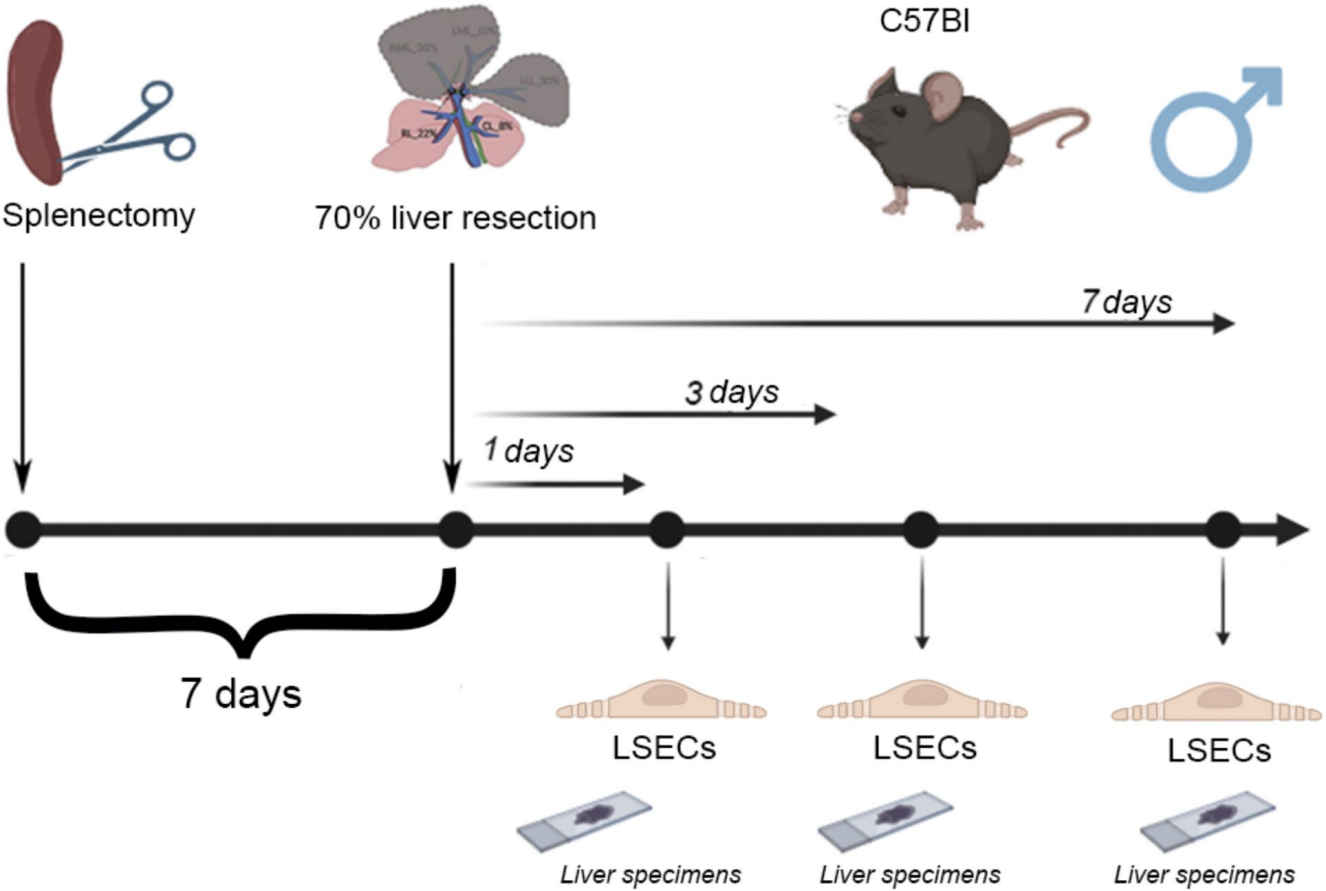
Scheme of the experiment.

The 70% liver resection (partial hepatectomy) in C57BL/6 male mice (n = 36) was performed under general isoflurane anesthesia using the Higgins and Anderson method^3^. The operations were carried out from 10.00 p.m. to 11.00 p.m. The animals were randomly assigned to two series of experiments: (1) partial hepatectomy in intact animals (n = 18); and (2) partial hepatectomy in animals splenectomized in advance (7 days before liver resection) (n = 18) using a previously described technique: the abdominal cavity was opened, the spleen was brought out into the wound, its vessels were ligated and crossed; the wound of the anterior abdominal wall was sutured layerwise (Fig. 1)^18^. The ventral incision was closed with suture and treated with 0.05% chlorhexidine bigluconate followed by an alcohol swabbing. The anterior abdominal wall was sutured in layers. The muscles and skin were sutured with separate knotted sutures (COATED VICRYL® (polyglactin 910) Suture, Ethicon, United States).

The figure was designed using the free version of the open-access online application BioRender (https://www.biorender.com/).

In the post-operative period, the mice were housed 2 animals per cage under conditions of controlled light and dark cycle with free access to food and water. The level of pain was assessed by activity, food, and water intake. Meloxicam (1.0 mg/kg/day) was used to provide analgesia in the pre- and postoperative period and was administered 30 min before surgery and every 12 h for 2 days after surgery. Postoperatively, mice showed signs of pain (decreased mobility, animals stopped drinking and eating, and piloerection was observed). However, these signs resolved within 2–3 h. Postoperative mortality averaged 15%, with the highest mortality rates observed on days 1 and 2 after liver resection.

The animals were withdrawn from the experiment 1, 3 and 7 days after liver resection using a CO_2_ chamber. SO—sham-operated animals with intact liver (n = 6) and SE—pre-splenectomized/non-hepatectomized animals (n = 6) were used as controls. The study was approved by the Ethics Committee of the A.P. Avtsyn Research Institute of Human Morphology (protocol No. 29 (5) dated November 8, 2021).

### Functional tests

Recovery of liver function was assessed by serum albumin concentration, alanine aminotransferase (ALT) and aspartate aminotransaminase (AST) tests (Cormay Diagnostics, Poland). The study was performed on a biochemical analyzer Cormay Accent-200 (Cormay Diagnostics, Poland) according to the manufacturer protocols.

### Histology and immunohistochemistry

Liver, lung and kidney fragments were fixed in 10% neutral formalin and subjected to standard histological dehydration, paraffin embedding and sectioning. The 5–8 μm sections were stained with hematoxylin and eosin for pathomorphological assessment.

For immunohistochemical tests, the tissue was placed in liquid nitrogen and cryosectioned at 5–8 μm thickness. The sections were incubated with primary antibodies for 12 h and FITC -conjugated secondary antibodies (1:200, ab97050, Abcam, UK,) for 1 h. Anti-Ki67 antibodies (1:100, ab15580, Abcam, UK); the nuclei were counterstained with 4′,6-diamidino-2-phenylindole (DAPI, Sigma-Aldrich Co LLC). The positivity indexes were determined as the percentage of labeled cells in total cell counts, with at least 3000 cells counted for each marker.

### Isolation of liver stromal cells

Under general anesthesia, the liver was perfused with a phosphate-buffered saline solution through the portal vein. The organ was then removed and weighed, washed twice with Hanks’ solution, crushed and incubated in a 0.1% solution of collagenase types 4 and 1 (PanEco, Russia) for 30 min at 37 °C on a horizontal shaker. The suspension was then filtered through a 100 μm nylon filter (SPL Life Science, Republic of Korea) and washed twice from the enzyme solution with centrifugation at 300 g for 10 min at 20 °C. After that, the cells were resuspended in 30 ml of phosphate-buffered saline and centrifuged for 3 min at 50 g. The centrifugation collected the parenchymatous liver cells (hepatocytes) in the sediment while leaving the non-parenchymatous elements including endothelial cells in the supernatant portion.

### Isolation of liver sinusoidal endothelial cells

The mechanical and enzymatic disaggregation of the liver into cell suspension was applied as described in the previous section. Percoll reagent (Percoll density gradient media—GE Healthcare Life Sciences) was used to separate the cells in a density gradient. A 25% Percoll solution was layered on top of a 50% Percoll solution in a 1:1 ratio. The liver cell suspension was layered in a 1:4 volume ratio relative to the gradient solution and the tube was centrifuged for 30 min at 1350 rpm in a smooth braking mode. The interphase ring containing cells of the non-parenchymal fraction, including endothelial cells, was collected, washed from Percoll by centrifugation for 15 min at 600 g (twice) and resuspended in a PBS buffer solution. After that, the cells were counted and their viability was assessed using a TC20 analyzer (Bio-Rad, USA). The cells were then subjected to immunomagnetic sorting on a manual MidiMACS™ Separator using LS Columns (MiltenyiBiotec, Germany) and using CD146 (LSECs) MicroBeads magnetic microparticles (MiltenyiBiotec, USA) in accordance with the manufacturer’s recommendations. The purity (using antibodies to CD146 and F4/80) and viability of isolated cells were checked. The viability of isolated cells before and after sorting was approximately 90% (Supplement Fig. 2). The proportion of CD146 + cells was approximately 90%, the proportion of F4/80 + cells was approximately 12% (Supplement Fig. 3).

### Flow cytometry

A 10^5^ aliquote of LSECs isolated using magnetic sorting for the CD146 marker were incubated in 100 µl Rinsing Solution (Miltenyi Biotec, USA) with 5 µl primary antibodies to CD31 (PECAM-1) (PE-labeled, 130–111-540, clone REA784 | 390, Miltenyi Biotec, USA), integrin alpha-5 (CD49e) (PE-labeled, 130–122-072, clone REA1183 | 5H10-27, Miltenyi Biotec, USA), VCAM-1 (CD106) (PE-labeled, 130–116-323, clone REA971 | 429, Miltenyi Biotec, USA), VE-cadherin (CD144) (PE-labeled, 130–128-207, clone REA225 | BV13, Miltenyi Biotec, USA), Ki67 (FITC-labeled, 130–117-691, clone REA183 | B56 Miltenyi Biotec, USA), CD146 (130–102-230, clone ME-9F1, Miltenyi Biotec, USA), F4/80 (130–102-422, REA126 | BM8, Miltenyi Biotec, USA ) at room temperature for 1 h.

The dynamics of CD3 + lymphocytes and NK1.1 cells in regenerating livers were analyzed similarly using 10^5^ cells of the liver stromal fraction and primary antibodies to CD45 (PE-labeled, 130–102-596, clone 30F11, Miltenyi Biotec, USA), CD3 (APC-labeled, MCA500APC, clone KT3, Bio-Rad USA), NK1.1 (VioBlue-labeled, Biolegend, USA); the incubations proceeded for 1 h.

Following the incubations, the cells were washed in PBS, resuspended in 0.5 ml PBS and analyzed in a MACSQuant 10 flow cytometer (Milteniy Biotech, Germany); the data were analyzed in FlowJo (LLC). Each measurement involved 10^4^ cells. The population of interest was defined in the frontal scatter (FSC) and side scatter (SSC) plots, excluding cellular debris. To determine the number of endothelial cells positive for a particular marker, we identified the positive population in dot plots of SSC and the corresponding marker (Supplement Fig. 4).

To determine the number of a particular lymphocyte population, we also determined the population of interest in the FSC-SSC plot, excluding debris. Then, in the SSC-CD45 dot diagram, the total population of CD45 + leukocytes was gated, and then in the CD3 + or NK1.1 + versus CD45 + cells dot diagram, the proportion of the corresponding cells was determined (Supplement Fig. 5).

### Apoptotic activity assay

The number of endothelial cells undergoing apoptosis was determined using the Annexin V + PI methods (TransDetect® Annexin V-FITC/PI Cell Apoptosis Detection Kit, Transgen Biotech, China).

### Transcriptomic assay

Total RNA was isolated from endothelial cells obtained by magnetic sorting for CD146 using the RNeasy Plus Mini Kit (QIAGEN, Germany). Transcriptome analysis was performed using microarray Clariom™ S Assay, mouse (Applied Biosystems™). Next, reverse transcription using the WT Amplification Kit was performed to synthesize first-, second-strand cDNA, cRNA and ss-cDNA incubating at appropriate temperatures and purifying via magnetic beads. Then, ss-cDNA was fragmented, labeled with biotin, hybridized to the array at 45 °C for 16 h, followed by washing. GeneChips were scanned using the Affimetrix GeneChip Scanner 3000 7 g. The data were analyzed with Transcriptome Analysis Console (TAC) Software version 4.0.2 using Affymetrix default analysis settings and RMA algorithm. Raw data is available in the BioStudies, S-BSST1922, https://www.ebi.ac.uk/biostudies/studies/S-BSST1922. Quality control data of all samples are presented in Supplement Fig. 7. Enrichment analysis was performed in TAC software and the Enrichк-KG service^19^. A fold change greater or less than 2 is used to select a gene for analysis. Only genes with a adjusted p-value less than 0.05 were included in the analysis.

### PCR assay

Total RNA obtained from endothelial cells isolated by magnetic sorting was used in cDNA synthesis with MMLV RT kit (Evrogen, Russia). The real-time PCR mixtures were set in duplicates by using qPCRmix-HS SYBR mastermixes (Evrogen, Russia). Gene-specific primers used in the assay are listed in Supplement Table 1. PCR primers for mRNA target. The relative expression levels were calculated using ΔCt approach against *Gapdh* as a reference transcript.

### Statistical analysis

The data were analyzed in SigmaStat 3.5 (Systat Software Inc, USA). Kolmogorov–Smirnov criterion was used to determine whether it conformed to normal distribution. In case of conformity to the normal law, the data were analysed using One Way ANOVA with the post-hoc Holm-Sidak test. In case of non-compliance with the normal law, the data were analyzed using ANOVA on ranks with the post-hoc Tukey test or Dunn’s method. The differences at *p* < 0,05 were considered significant. Paired comparisons used the Student’s t-test for normal distributions and the Mann–Whitney U-test for distributions other than normal.

## Results

### Effect of splenectomy on the dynamics of liver regeneration

**Fig. 2:**
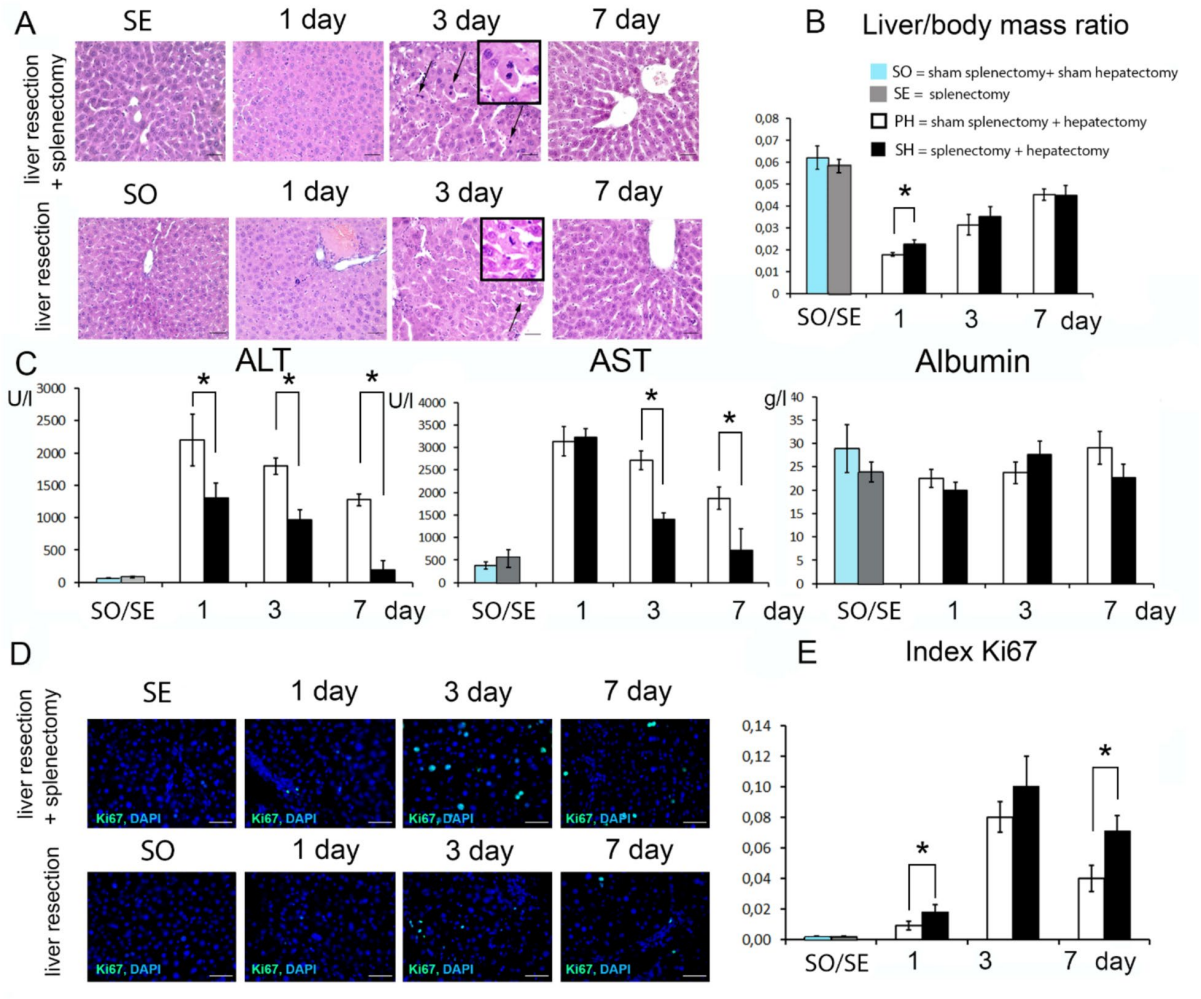
Characteristics of liver regeneration after 70% resection under conditions of previous splenectomy. (**A**) Histological structure of the regenerating liver, hematoxylin and eosin stain, scale bar—100 µm, arrows indicate mitotic figures in hepatocytes. (**B**) Dynamics of liver mass recovery after 70% resection. (**C**) Serum concentrations of ALT, AST and albumin in operated mice. (**D**) Immunohistochemical study of the proliferation marker Ki67 in the regenerating liver. Second antibodies are conjugated to FITC (green), nuclei are counterstained with DAPI (blue), scale bars—50 μm. (**E**) Dynamics of the Ki67 index. Data are presented as mean ± SD, SO – sham splenectomy + sham hepatectomy (n = 6), SE—splenectomy, (n = 6), SH – splenectomy + hepatectomy (n = 18), PH—sham splenectomy + hepatectomy, *—statistically significant differences, *p* < 0,05. Paired comparisons used the Student’s t-test for normal distributions and the Mann–Whitney U-test for distributions other than normal.)

Splenectomy did not cause any noticeable changes in the liver structure of experimental animals. The parenchyma was represented by hepatic lobules consisting of hepatocyte cords. Portal tracts were located on the periphery of the lobules (Fig. 2 A). On the contrary, liver resection induced significant changes, as manifested by the appearance of a large number of mitotic figures. The greatest number of mitotic figures was found after 3 and 7 days, both in the liver of animals with preliminary splenectomy and preserved spleen (Fig. 2 A). Restoration of liver mass, the main indicator of the post-resection liver recovery, is complete in about 7 days post-resection (Fig. 2 B).

The absence of the spleen affected the dynamics of liver mass restoration in experimental animals. It was found that the liver weight of animals with removed spleen was significantly higher compared to animals with preserved spleen 1 day after liver resection (*p* < 0,024, Fig. 2 B, Supplement Table 3).

These data were consistent with the dynamics of biochemical parameters (Fig. 2 C, Supplement Table 4). Throughout all terms of the study the ALT level was significantly higher in blood plasma of animals with preserved spleen (*p* < 0,018, *p* < 0,001, *p* < 0,029 for 1, 3 and 7 day, respectively). In addition, it was found that AST activity was significantly higher in the blood of animals with preserved spleen on 3 and 7 days after liver resection (*p* < 0,001 and *p* < 0,029, respectively, Fig. 2 C, Supplement Table 4). Albumin concentration did not differ between animals with preserved spleen and splenectomy (Fig. 2 C, Supplement Table 4).

The more rapid recovery of liver weight in splenectomized animals was based on a more pronounced proliferative response to resection of liver parenchyma. In animals splenectomized 7 days before the resection, Ki67 + cell index of the liver remnant was significantly higher (Fig. 2 D). Statistically significant differences were found 1 and 7 days after liver resection (*p* < 0,001 and < 0,001, respectively, Fig. 2 E, Supplement Table 5).

Animals with and without prior splenectomy showed no pathological changes in the lungs or kidneys after liver resection. In all cases, the histological structure characteristic of the lungs and kidneys, respectively, was observed (Supplement Fig. 1). The surgical interventions performed did not significantly affect the body weight of the operated animals (Supplement Table 2).

### Effects of splenectomy on liver sinusoidal endothelial cell immunophenotypes

**Fig. 3:**
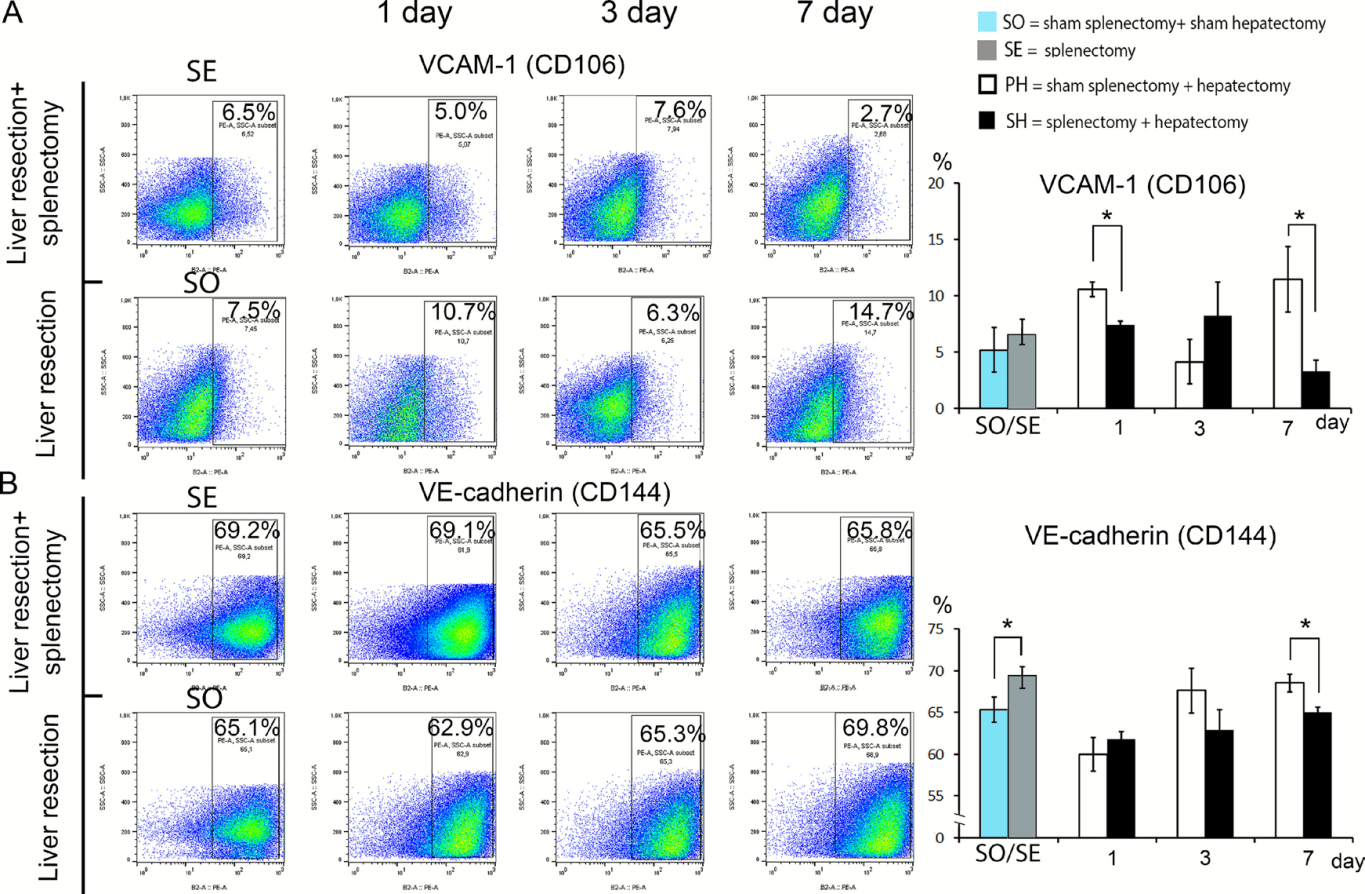
Characterization of immunophenotype of endotheliocytes of sinusoidal capillaries of regenerating liver. (**A**) Dynamics of the population of VCAM-1 + cells in the regenerating liver. (**B**) Dynamics of the population of VE-cadherin + cells in the regenerating liver. Data are presented as mean ± SD, SO – sham splenectomy + sham hepatectomy (n = 6), SE—splenectomy, (n = 6), SH – splenectomy + hepatectomy (n = 18), PH—sham splenectomy + hepatectomy, *—statistically significant differences, *p* < 0,05. Paired comparisons used the Student’s t-test for normal distributions and the Mann–Whitney U-test for distributions other than normal.

Splenectomy affected the state of endothelial cells of the sinusoidal capillaries of the regenerating liver. The most pronounced dynamics were observed for cells positive for VCAM-1 protein (CD106) (Fig. 3A, Supplement Table 6). The preceding removal of the spleen led to a statistically significant decrease in the number of CD106 + cells in the liver on days 1 and 7 post-hepatectomy (*p* < 0,005 and < 0,035, respectively, Fig. 3A, Supplement Table 6). In addition, a significant decrease in VE-cadherin-positive (CD144 +) endothelial cells was observed in animals of the preceding splenectomy group on post-hepatectomy day 7 (Fig. 3B, p < 0,007, Supplement Table 6). A tendency towards a decrease in PECAM-1-expressing (CD31 +) cell counts was noted (p = 0.062, Supplement Table 6), while counts of integrin alpha-5-positive (CD49e +) cells did not change significantly (Supplement Fig. 6, Supplement Table 6).

### Cell death levels and proliferative activity of liver sinusoidal endothelial cells

**Fig. 4:**
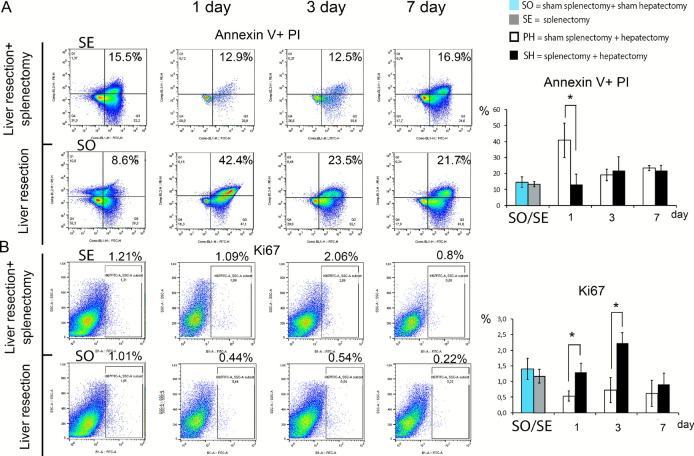
Activity of cell death and proliferation of endotheliocytes of sinusoidal capillaries of regenerating liver. (**A**) Dynamics of cell death in the regenerating liver. (**B**) Dynamics of the population of Ki67 + endotheliocytes in the regenerating liver. Data are presented as mean ± SD, SO – sham splenectomy + sham hepatectomy (n = 6), SE—splenectomy, (n = 6), SH – splenectomy + hepatectomy (n = 18), PH—sham splenectomy + hepatectomy, *—statistically significant differences, *p* < 0,05. Paired comparisons used the Student’s t-test for normal distributions and the Mann–Whitney U-test for distributions other than normal.

Liver resection without splenectomy caused an increase in the relative counts of dying endothelial cells in the remnant. On day 1 post-hepatectomy, the number of endothelial cells in apoptosis was significantly higher in the group of animals with preserved spleen (*p* < 0,019, Fig. 4A, Supplement Table 6). The preceding splenectomy affected the proliferative activity of sinusoidal capillary endothelial cells: the Ki67 + index was significantly higher in the group of animals with removed spleen on day 1 and 3 (*p* < 0,019 and < 0,029, respectively, Fig. 4B, Supplement Table 6).

### Lymphocyte population dynamics in regenerating livers

**Fig. 5:**
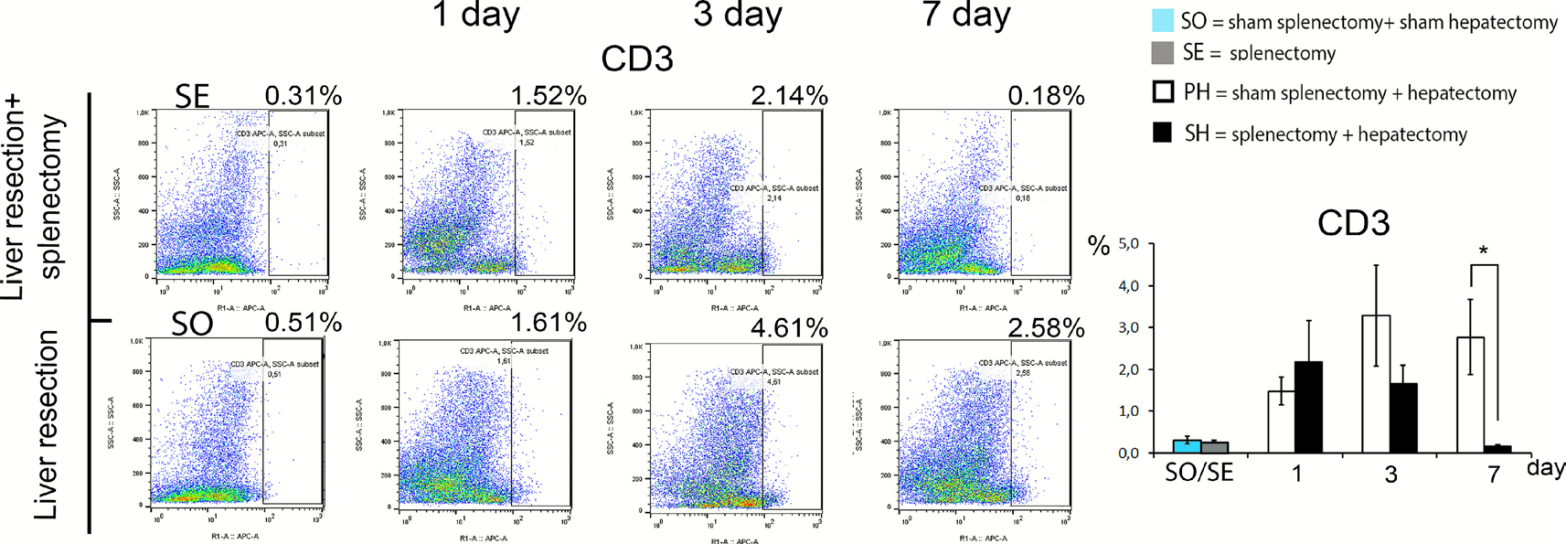
Dynamics of the CD3 + cells in the regenerating liver. Data are presented as mean ± SD, LR – 70% liver resection, SO – sham splenectomy + sham hepatectomy (n = 6), SE—splenectomy, (n = 6), SH – splenectomy + hepatectomy (n = 18), PH—sham splenectomy + hepatectomy,*—statistically significant differences, *p* < 0,05. Paired comparisons used the Student’s t-test for normal distributions and the Mann–Whitney U-test for distributions other than normal.

On the 7th day after liver resection, a decrease in the number of CD3 + cells in the regenerating liver of animals that had previously undergone splenectomy was found (*p* < 0,011, Fig. 5, Supplement Table 7). The number of NK1.1-cells in intact and regenerating liver is extremely low, which makes it difficult to statistically analyze the data obtained. There was no difference when comparing the relative value of the share of NK1.1-cells from the number of CD3 + lymphocytes. However, taking into account the decrease of CD3 + cells in splenectomized animals there is also a decrease in the number of NK1.1-cells on day 1 and 7 after hepatectomy. This is noticeable by absolute values (*p* < 0,009 and *p* < 0,018, respectively, Supplement Table 8).

### Transcriptome analysis of sinusoidal endothelial cells in regenerating livers

**Fig. 6:**
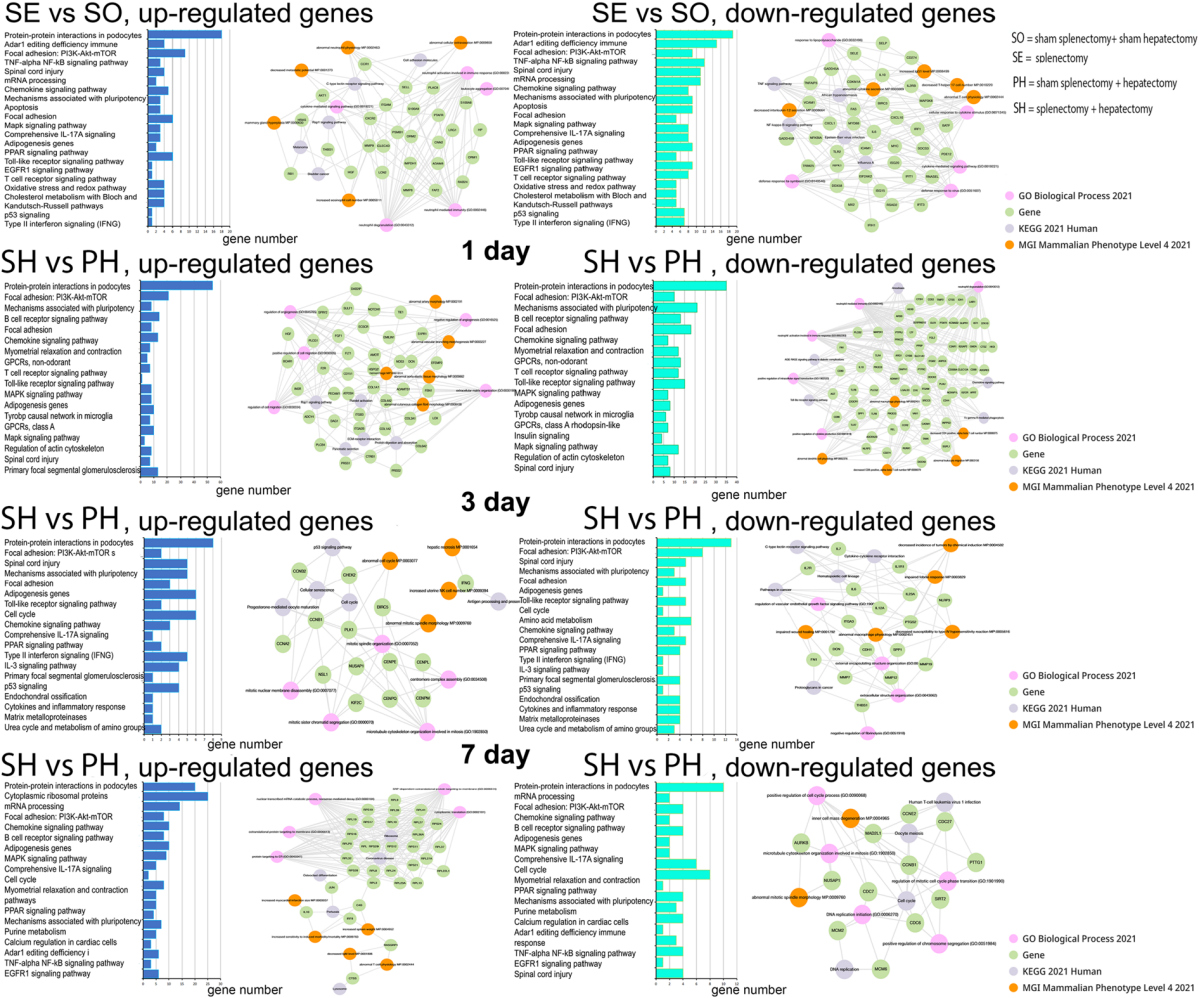
Analysis of the transcriptome of endotheliocytes in the regenerating liver. SO – sham splenectomy + sham hepatectomy (n = 6), SE—splenectomy, (n = 6), SH – splenectomy + hepatectomy (n = 18), PH—sham splenectomy + hepatectomy, (n = 3).

The preceding splenectomy affected gene expression profiles of the sinusoidal capillary endothelial cells in both intact and regenerating liver as was shown by microarray analysis (Fig. 6). Principal component analysis (PCA) based on transcriptome data revealed interesting patterns: endothelial samples from combined splenectomy and liver resection grouped more closely (especially on days 1 and 3) than those from hepatectomy alone (Supplement Fig. 8). The same was true for sham-operated animals and only splenectomy samples on day 0, which practically formed a single group.

In the intact liver after splenectomy, 694 differentially expressed genes were detected compared to the liver of animals without splenectomy. Among the most enriched were pathways including PI3K-Akt-mTOR signaling, Apoptosis, Chemokine signaling and Focal adhesion. We then conducted enrichment analysis separately for genes with increased and decreased expression. Splenectomy induced activation of pathways associated with extravasation, neutrophil activation, and leukocyte aggregation in endothelial cells (Fig. 6, Day 0). Among suppressed genes we found the signaling pathways associated with the inflammatory response (including response to lipopolysaccharide, NF-kappa B signaling, and TNF signaling) and cytokine response were identified (Fig. 6, Day 0).

On post-hepatectomy day 1, 1086 differentially expressed genes were detected for LSECs of animals with vs without splenectomy. It is interesting to note the *Hgf* and *eNOs* genes among those with increased expression. Among all genes following pathways were again detected: PI3K-Akt-mTOR signaling, Focal adhesion and Chemokine signaling pathway, as well as inflammation-related pathways B cell receptor signaling pathway, T cell receptor signaling pathway, Toll-like receptor signaling pathway. As for separate analysis for up-regulated genes, on post-hepatectomy day 1, pathways associated with the regulation of angiogenesis, cell migration, and extracellular matrix organization were activated (Fig. 6, Day 1). Neutrophil- and macrophage-associated signaling pathways were down-regulated compared to hepatectomy samples in the same samples (Fig. 6, Day 1).

By post-hepatectomy day 3, the number of differentially expressed genes decreased to 437. PI3K-Akt-mTOR signaling pathway and Focal adhesion pathway were still found among the most enriched signaling pathways among all up- and down-regulated genes. The genes with increased expression at this time point included cell cycle-associated genes (*Ccna2*, *Ccnb2*, etc.), which was reflected in the list of the most enriched signaling pathways, notably the Cell cycle and mitosis. P53 protein, hepatic necrosis and cellular senescence associated pathways were also found among up-regulated genes (Fig. 6, Day 3). Pathways associated with hematopoiesis, wound healing, endothelial and macrophage growth signaling were suppressed at day 3 post-resection in samples with splenectomy compared to samples without it, but with hepatic resection (Fig. 6, Day 3).

By post-hepatectomy day 7, for animals with and without preceding splenectomy, the number of differentially expressed genes in LSECs was 726. The most enriched signaling pathways among all up and down-regulated genes included the Focal adhesion: PI3K-Akt-mTOR signaling pathway, Cell cycle, Chemokine signaling pathway. Pathways associated with translation, ribosomes, protein targeting to the ER and increased spleen weight were activated among up-regulated genes (Fig. 6, Day 7). Signaling pathways associated with the cell cycle, DNA replication, and mitosis were suppressed in similar samples among down-regulated genes (Fig. 6, Day 7).

The data obtained from the transcriptome analysis of isolated LSECs were generally confirmed by gene expression analysis using real-time PCR. It was found that in animals whose spleen was removed 7 days before 70% liver resection, the expression of proinflammatory cytokines and chemokines was significantly lower compared to animals with preserved spleen (Supplement Table 9). The most pronounced effect was observed in the early stages after liver resection. Lower expression after liver resection was observed for the following genes: *Il1β, Il6, Cx3cr1, Ccr2, Ccl6, Ccl9, Ccl4, Ccr1* (Supplement Table 9). Moreover, for some genes, a lower expression of the studied genes was noted in LSECs in animals that underwent only splenectomy, compared to animals in which both the spleen and liver were intact. These genes include the following: *Il6 (p* < 0,046)*, Ccr2 (p* < *0,001), Ccl4 (p* < *0,001) and Ccr1 (p* < *0,001)* (Supplement Table 9).

## Discussion

Liver sinusoidal endothelial cells (LSECs) constitute approximately 15–20% of all liver cells, but account for less than 3% of the organ volume^20^. The sinusoidal endothelium is a key regulator of liver regeneration as it balances the hepatocyte proliferation with the vascular density^6^. After resection, the hemodynamic conditions in the liver change dramatically, which some authors consider to be essential for the onset of liver regeneration^2^. An increase in the relative intensity of the portal blood flow through the liver initially affects LSEC functioning. Under the altered hemodynamic conditions, the endothelial cells release nitric oxide (NO) which increases the hepatocyte sensitivity to HGF^21^, downregulate the TGFβ1 signaling capacity^22^, and activate the HGF synthesis^23^.

A connection between the spleen and the liver has been established clinically, with splenomegaly and signs of hypersplenism associated with pathological conditions of the liver^9^. Splenectomy has been proposed as the means to relieve the hypersplenism manifestations typical under these conditions^9,10^. The removal of the spleen has been shown to exert a beneficial effect on the state of fibrotic liver^24^ or the liver after large-scale resection^15^, as well as to improve the liver transplant engraftment^14^. In the current study, this phenomenon was also confirmed in a liver resection model. We have shown more effective recovery of liver mass. The mechanisms of this phenomenon are studied insufficiently. Resection of extremely large liver volumes, similarly with profound fibrotic changes to the organ, have been associated with portal hypertension and corresponding damage to the endothelial cells of liver sinusoids^25^. Under these conditions, potentially, removal of the spleen substantively relieves the portal blood flow rates with an overall beneficial effect on the state of the endothelial cells of the sinusoidal capillaries of the liver. This has been shown in a model of liver fibrosis in laboratory animals, as well as in patients with liver cirrhosis^26,27^.

Despite the theoretical background, the role of sinusoidal capillary endothelial cells in the therapeutic effect of splenectomy on the liver under various pathological conditions remains unclear^17,28^. In this study, we address this issue using a model of liver regeneration after 70% resection. Based on the data, it can be concluded that splenectomy affects the state of the sinusoidal capillary endothelial cells in the regenerating liver. The effects of splenectomy include the alleviated signs of the so-called shear stress of endothelial cells and the decreased apoptotic cell counts, consistent with the lower Ki67 + LSEC counts, as well as the reduced signs of the endothelial cell inflammatory activation.

The effect of shear stress on endothelial cells is mediated by cell junction proteins that implement mechanosensing^29,30^, including PECAM-1, VCAM-1 and VE-cadherin^29,30^. The reduced counts of PECAM-1 and VCAM-1 positive endothelial cells in the liver of animals that had their spleen removed before liver resection indicate a less pronounced shear stress in LSECs under conditions of preceding splenectomy. However, it should be noted that, judging by the expression profile of endothelial cells, the changes were not so unambiguous. Along with genes with reduced expression, a large number of genes with increased activity were found, which also relate to the Focal adhesion signaling pathways: PI3K-Akt-mTOR signaling pathway, Focal adhesion. In this regard, it cannot be said that splenectomy completely neutralizes the damaging effect of portal hypertension on LSECs.

In addition, one cannot exclude the influence of the spleen as a source of synthesis of proinflammatory cytokines, activated in response to an increase in the concentration of LPS in the blood and the entry into the bloodstream of liver cell death products, which can also activate the endothelial cells of the liver sinusoidal capillaries. LSECs are known to participate in the development of acute inflammatory liver disorders, particularly in connection with their involvement in the migration of leukocytes, as well as the metabolization of endotoxin^31^ and most IgG immune complexes from the blood^32^. In addition, the inflammation is accompanied by increased rates of VCAM-1 and PECAM-1 synthesis, which facilitates the crossing of the sinusoidal vascular walls by leukocytes^20^. Under physiological conditions, LSECs that act as antigen-presenting cells can induce the development of immunological tolerance in CD8 + T cells^33^, and also promote the differentiation of T cells into immunosuppressive lymphocytes^34^.

The data we obtained are consistent with the cited studies. In this regard, in our opinion, the most pronounced effect of splenectomy is achieved by reducing inflammation in the liver, and one of the leading roles in this process also belongs to LSECs. Molecular cascades related to inflammation, migration and activation of leukocytes were enriched in the endothelial cells of the regenerating liver of animals with preceding splenectomy, including the Chemokine signaling pathway, B cell receptor signaling pathway, T cell receptor signaling pathway and Toll-like receptor signaling pathway. Noteworthy, among the differentially expressed genes of these signaling pathways, genes with reduced expression prevailed (especially on post-resection day 1).

Noteworthy, splenectomy was shown to improve the liver transplant engraftment^14^. Considering the role of T cells in transplant rejection, it is reasonable to link the beneficial effect to the splenic T cell pools withdrawal. This hypothesis supported by our data on the reduced T cell and NK1.1 cell counts in regenerating liver of splenectomized animals, as well as by the transcriptome analysis data.

Overall, different lymphocyte subsets affect liver regeneration in specific patterns. For instance, γδT cells secrete IL-17^35^ which supports IL6 synthesis and inhibits IFN-γ synthesis by liver macrophages thereby stimulating liver regeneration^36^. The CD49a + innate lymphoid cells also found in the liver produce high amounts of NKG2A which prevents the NK cell migration to the liver^37^. The NK-, NKT- and NK1.1 cells inhibit liver regeneration as suggested by the IFN-γ synthesis^38,39^. The observed reduction in the counts of IFN-γ-secreting cells, exemplified by NK1.1 populations, may therefore indicate a positive effect of splenectomy on liver regeneration.

Based on the findings, it can be assumed that the stimulating effect of splenectomy on the repair processes in the liver after its extensive resection or transplantation is achieved through inhibition of the proinflammatory activation of LSECs and the associated migration of leukocytes to the liver^18,40^, including the lymphocyte subsets that inhibit liver regeneration, such as CD161 + cells. Thus, splenectomy appears to prevent the inflammatory damage to the regenerating liver. The effect involves two distinct mechanisms. First, splenectomy removes an essential source of migratory granulocytes, monocytes^40^ and (as demonstrated by the current evidence) lymphocytes to the liver. Second, the spleen may act as a source of various pro-inflammatory cytokines and the removal of this source prevents the pro-inflammatory activation of LSECs which further alleviates the migration of leukocytes to the liver. In the context of liver resection, the potential for a reduction in portal hypertension after splenectomy cannot be excluded.

The prospects for further developments in this area open up new horizons for clinical practice and research. First, additional studies are needed to better understand the molecular and cellular mechanisms underlying the observed effects of splenectomy which include analysis of specific cytokines and signaling pathways. Second, it is important to investigate the effects of splenectomy on different leukocyte subsets and their role in liver regeneration. Understanding these processes may lead to the development of new therapeutic strategies aimed at modulating the immune response in liver diseases. Third, alternative methods such as pharmacological blockade of pro-inflammatory pathways should be considered, which may be a less invasive alternative to splenectomy. This may open up new treatment options for patients with severe liver pathologies where splenectomy may be contraindicated. Thus, further research in this area could lead to the development of new, more effective treatments for liver diseases, which will ultimately improve the quality of life of patients.

Furthermore, the impact of splenectomy on immune status and peripheral blood parameters remains an important question, as expected. Clinical observations indicate that after spleen removal, a number of patients develop so-called postsplenectomy syndrome, which is manifested by a predisposition to infectious diseases^41^. In some cases, a predisposition to malignant neoplasms has been demonstrated. However, the time frame for the development of these complications varies across studies^42^. One study indicates a period of 2 to 10 years^43^. This is consistent with studies conducted on laboratory animals. Thus, after splenectomy in mice, the number of lymphocytes and granulocytes in the blood does not change significantly for 2 months after surgery; changes were detected only after 8 months^44^. However, after 2 months, an increase in the number of CD19 + B lymphocytes was detected, while after 8 months, the number of CD3 + and CD19 + cells significantly decreased^45^. An increase in the number of platelets was detected 6 months after splenectomy^46^.

## Limitations of the study

This study has several limitations that should be acknowledged. The question of LSECs participation in liver regeneration is quite complex. However, none of the researchers doubts the key role of LSECs in this process^47^. In general, endothelial cells can act as sensors of changes in the hemodynamic level in the liver, which are observed after its resection. It has been shown that after liver resection, endothelial cells increase NO production, under the influence of which hepatocytes become more sensitive to HGF^21^. On the other hand, LSECs can act as a source of synthesis of a number of cytokines that stimulate liver regeneration. For example, the synthesis of IL6^48^, which provides the priming of hepatocytes before they enter the mitotic cycle, as well as the synthesis of HGF, the main mitogen for hepatocytes^23^.

Based on the obtained data, the beneficial effect of splenectomy on liver regeneration mediated by LSECs is achieved through the following mechanisms:

1. Increased synthesis of HGF and NO by LSECs. We found increased expression of the eNOs gene and HGF gene in LSECs 1 day after liver resection in splenectomized animals.

2. Splenectomized animals have more effective proliferation of LSECs, which in turn ensures more active proliferation of hepatocytes and restoration of liver mass.

3. Splenectomized animals lack proinflammatory activation of LSECs, which prevents migration of lymphocytes (CD3 + , NK1.1-cells) to the liver, inhibiting its regeneration after resection.

It is difficult to estimate the contribution of HGF synthesis by LSECs to liver regeneration, because even without HGF synthesis in the liver, its regeneration does not stop completely^49^. The same can be said about eNOs. It has been shown that in knockouts of this gene, liver regeneration after resection is inhibited, and an increase in the expression of the corresponding gene and the synthesis of eNOs^50^, on the contrary, stimulates liver regeneration. Thus, even a small increase in the expression of the *Hgf* and *eNOs* genes and the synthesis of the corresponding proteins can have a stimulating effect on liver regeneration^51^. However, further studies are required for this.

Another limitation is that the LSE samples were found to contain Kupffer cells, which likely affected the transcriptome analysis results.

## Supplementary Information

Supplementary Information.

## Data Availability

The original contributions presented in the study are included in the article/Supplementary Material, further inquiries can be directed to the corresponding authors. Raw data is available in the BioStudies, S-BSST1922, https://www.ebi.ac.uk/biostudies/studies/S-BSST1922.
