# Supplementary material for: Splenectomy reduces shear stress and inflammation in liver endothelial cells during regeneration after partial hepatectomy in mice
Source: Sci Rep. 2025 Dec 13;16:2706. doi: 10.1038/s41598-025-32446-4 (PMC12824239; doi:10.1038/s41598-025-32446-4)
Supplement: Supplementary file 1 — Supplementary Information. [file 41598_2025_32446_MOESM1_ESM.pdf]

Splenectomy reduces shear stress and inflammation in liver endothelial cells during regeneration after partial hepatectomy in mice

Andrey Elchaninov[1,3]*, Elena Gantsova[1,3], Polina Vishnyakova[2,3], Maria Kuznetsova[4], Dmitry Trofimov[4], Timur Fatkhudinov[1,3], Gennady Sukhikh[2]

1 Laboratory of Growth and Development, Avtsyn Research Institute of Human Morphology of FSBI "Petrovsky National Research Centre of Surgery", Moscow, Russia

2 Laboratory of Regenerative Medicine, Institute of Translational Medicine, National Medical Research Centre for Obstetrics, Gynecology and Perinatology Named after Academician V.I. Kulakov of Ministry of Healthcare of Russian Federation, Moscow, Russia

3 Research Institute of Molecular and Cellular Medicine, Peoples' Friendship University of Russia (RUDN University), Moscow, Russia

4 Laboratory of molecular research methods, Institute of Reproductive Genetics, National Medical Research Centre for Obstetrics, Gynecology and Perinatology Named after Academician V.I. Kulakov of Ministry of Healthcare of Russian Federation, Moscow, Russia

* Correspondence:
Andrey Elchaninov
elchandrey@yandex.ru

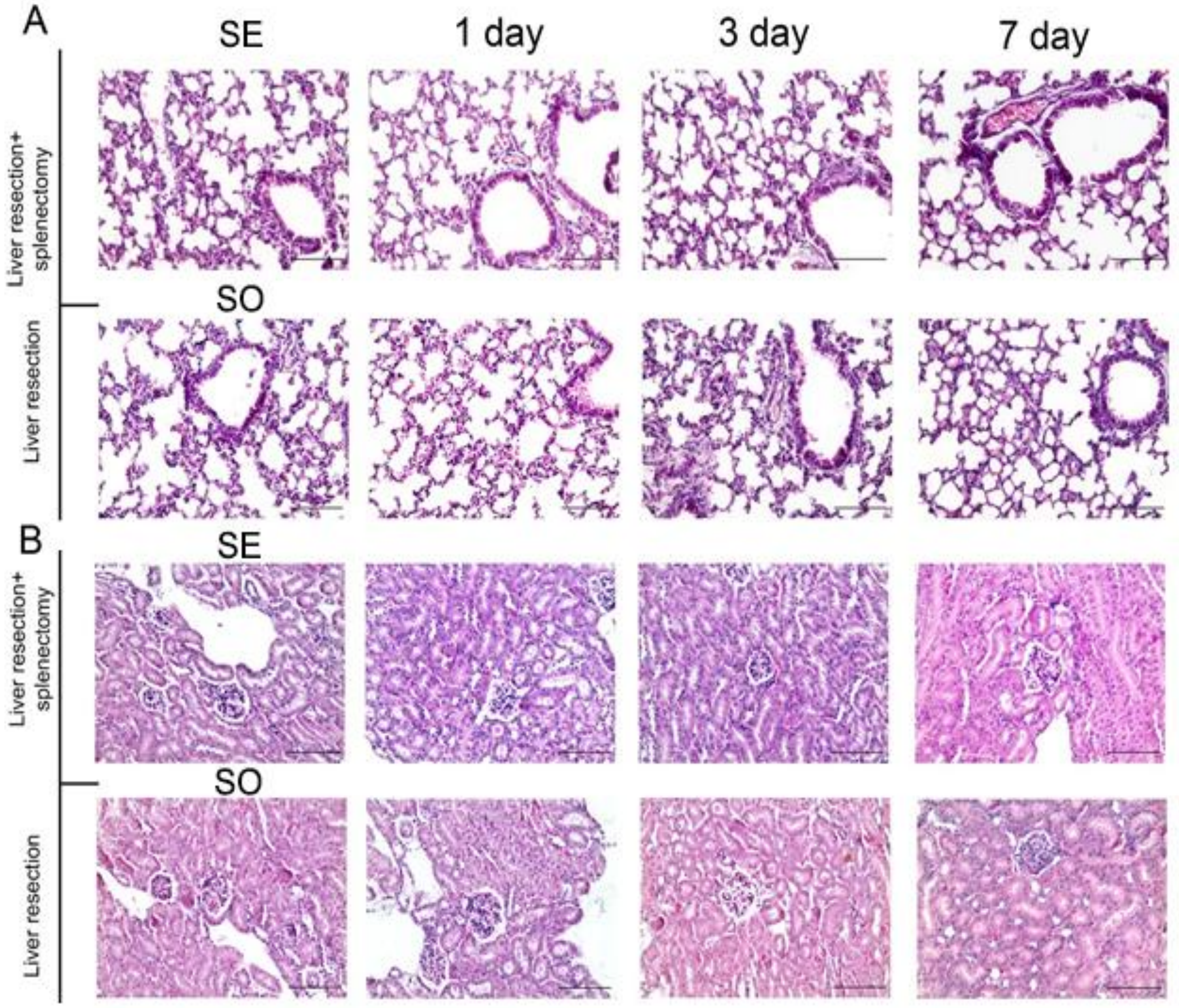

Supplement Figure 1. Histological structure of the lungs and the kidneys, hematoxylin and eosin staining, scale bar - 100 μm. SO – sham splenectomy + sham hepatectomy (n=6), SE - splenectomy, (n=6).

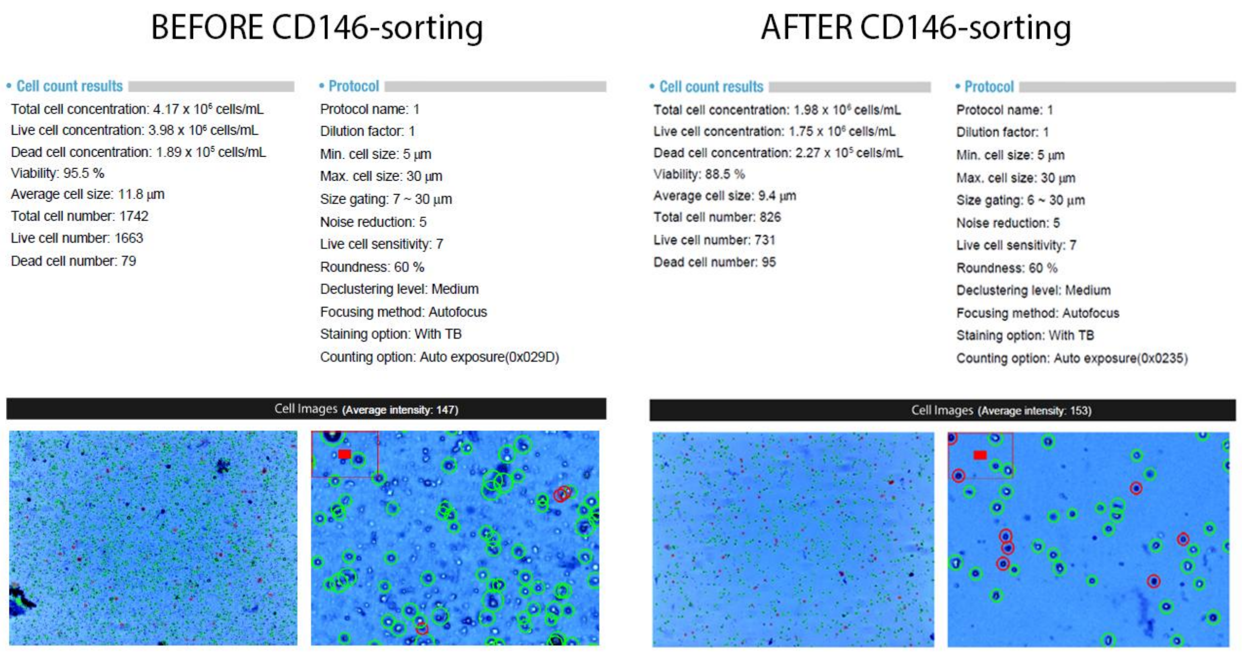

Supplement Figure 2. Evaluation of the viability of isolated cells.

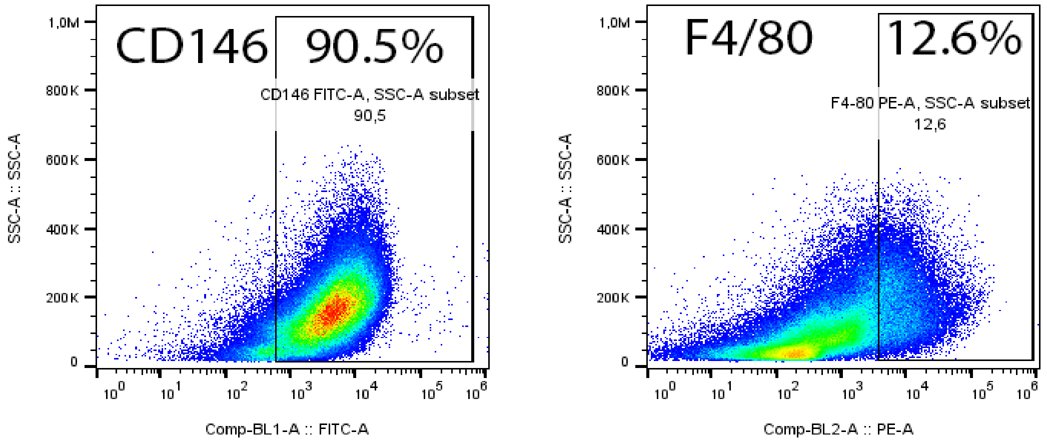

Supplement Figure 3. Evaluation of the purity of sorted LSECs. The proportions of CD146+ and F4/80+ cells are noted.

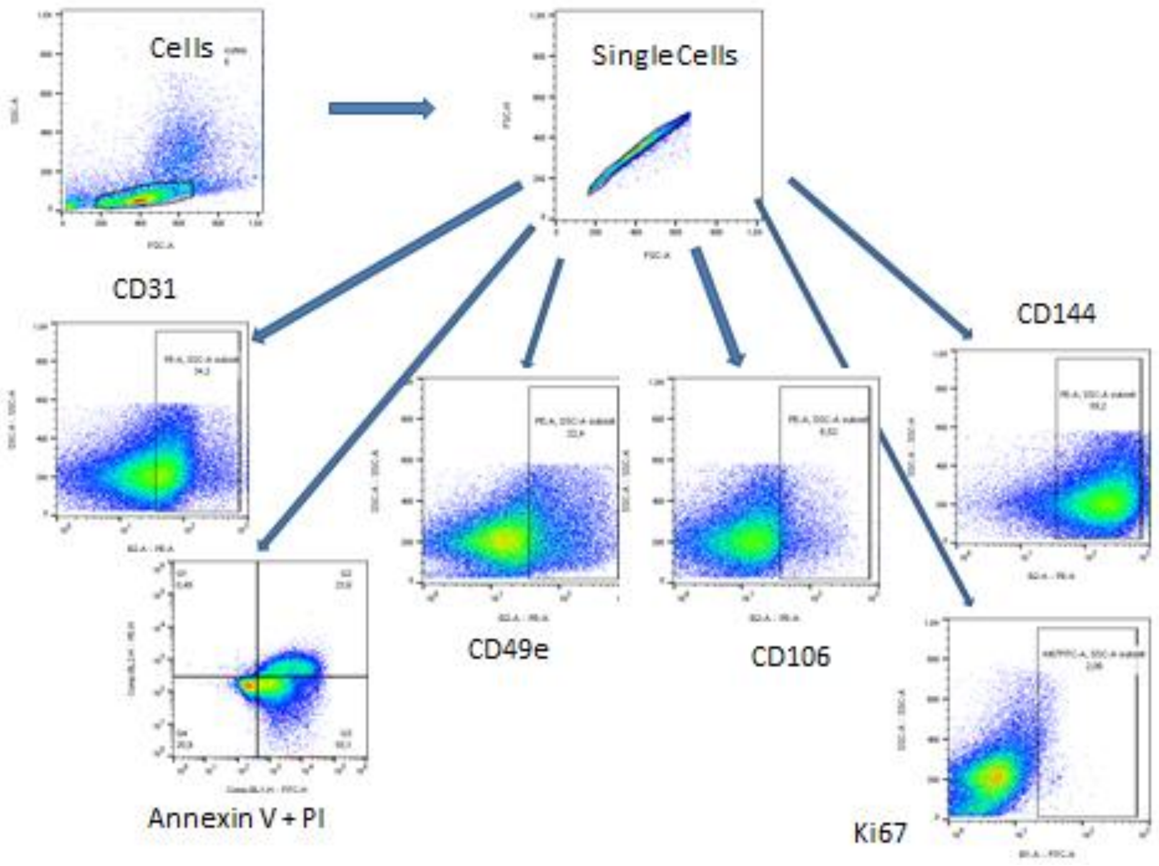

Supplement Figure 4 LSECs Gating strategy

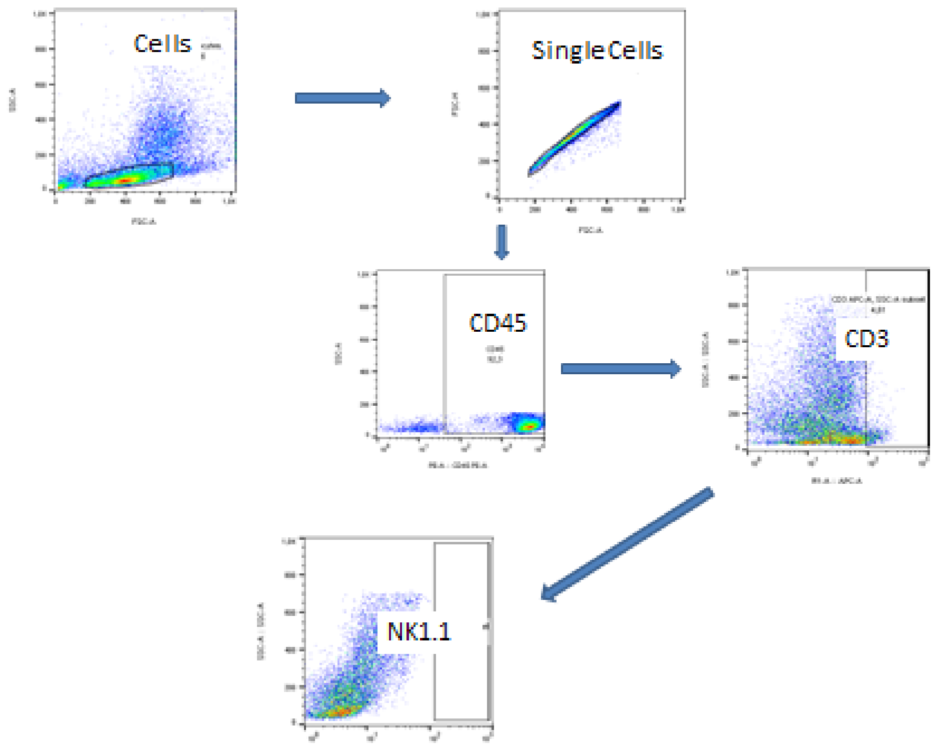

Supplement Figure 5 Lymphocytes Gating strategy

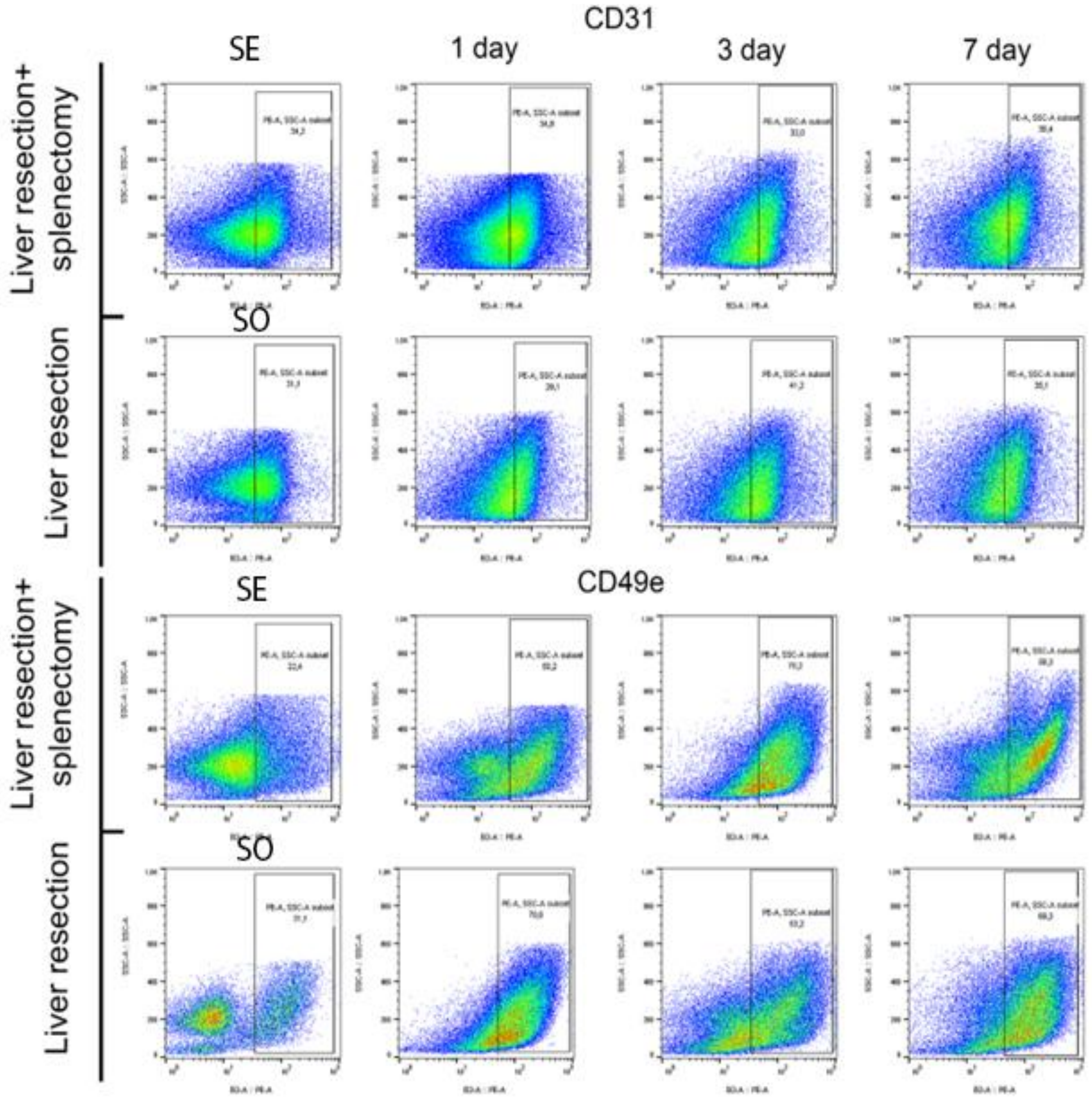

Supplement Figure 6. Dynamics of CD31+ and CD49e+ cells in the regenerating liver. Flow cytometry. SO – sham splenectomy + sham hepatectomy (n=6), SE - splenectomy, (n=6).

Supplement Table 1. List of primers

№	mRNA	Forward	Revers
1	*mIL1b-for*	CCTGAACTCAACTGTGAAATGC	GAAAGACACAGGTAGCTGCCA
5	*mIL6_n1-for*	TGCCTTCTTGGGACTGATGCT	TCTCTGGGTTGGCACACACT
8	*mCx3cr1_*	TCTGCGTGAGACTGGGTGAG	GAGGGCGTAGAAGACGGACA
9	*mCcr2_*	AGGGCATTGGATTCACCACA	GGCAGGATCCAAGCTCCAATTT
10	*mCcl6_*	TATCCTTGTGGCTGTCCTTGGG	GTGTGGCATAAGAGAAGCAGCAG
11	*mCcl9*	GCCCAGATCACACATGCAAC	CTGGAACCCCCTCTTGCTGAT
12	*mCcl4*	TTCCTGCTGTTTCTCTTACACCT	CTGTCTGCCTCTTTTGGTCAG
13	*mCcr1*	CTCATGCAGCATAGGAGGCTT	ACATGGCATCACCAAAAATCCA
14	*GAPDH*	AGGCCGGTGCTGAGTATGTC	TGCCTGCTTCACCACCTTCT

Supplement Table 2. Dynamics of the experimental animals body weight (BW)

Day	**SO, BW, mean ± SE, g**	**SE, BW, mean ± SE, g**	***p*-value**
**0**	19.0±1,4	17.0±1.082	0.4
	**PH, BW, mean ± SE, g**	**SH. BW, mean ± SE, g**	***p*-value**
**1**	18.0±0,8	21.7±5.131	1.0
**3**	19.8±1,5	17.9±0.41	0.8
**7**	19.7±0,6	20,6±2.552	1.0
SO – sham splenectomy + 70% liver resection (n=6), SE - splenectomy + 70% liver resection (n=6), SH – splenectomy + hepatectomy (n=18), PH - sham splenectomy + hepatectomy.			

Supplement Table 3. Dynamics of the liver-to-body weight ratio

Day	**SO, mean ± SE**	**SE, mean ± SE**	***p*-value**
**0**	0.062±0.003	0.0581±0.00008	0.533
	**PH, mean ± SE**	**SH, mean ± SE**	***p*-value**
**1**	0.0178±0.0004	0.0225±0.0013	**0.024***
**3**	0.0314±0.003	0.0317±0.003	0.952
**7**	0.0452±0.002	0.0447±0.00328	1.000
SO – sham splenectomy + 70% liver resection (n=6), SE - splenectomy + 70% liver			

resection (n=6), SH – splenectomy + hepatectomy (n=18), PH - sham splenectomy + hepatectomy, *- statistically significant differences, p<0.05

Supplement Table 4. Dynamics of biochemical parameters

**ALT**, U/l			
**Day**	**SO, mean ± SE**	**SE, mean ± SE**	***p*-value**
**0**	60.9±4.2	76.1±10.5	0.165
	**PH, mean ± SE**	**SH, mean ± SE**	***p*-value**
**1**	2205.3±231.1	1310.5±12.4	**0.018***
**3**	1798.3±44.9	70.4±2.1	**<0.001***
**7**	1275.5±43.6	192.75±72.8	**0.029***
**AST**, U/l			
**Day**	**SO, mean ± SE**	**SE, mean ± SE**	***p*-value**
**0**	377.4±39,1	551.7±130.4	0.267
	**PH, mean ± SE**	**SH, mean ± SE**	***p*-value**
**1**	3136.9±186.5	3222.7±65.2	0,750
**3**	2714.3±83.502	410.2±58.6	**<0.001***
**7**	1874.0±24.8	712.0±243.9	**0.029***
**Albumin**			
**Day**	**SO, mean ± SE**	**SE, mean ± SE**	***p*-value**
**0**	28.9±2.5	20.9±0.71	0,109
	**PH, mean ± SE**	**SH, mean ± SE**	***p*-value**
**1**	22.5±1.1	18.8±0.055	0.08
**3**	23.7±1.3	47.6±16.15	0.2
**7**	29.0±2.4	22.6±2.095	0.3
SO – sham splenectomy + 70% liver resection (n=6), SE - splenectomy + 70% liver resection (n=6), SH – splenectomy + hepatectomy (n=18), PH - sham splenectomy + hepatectomy, *- statistically significant differences, p<0.05			

Supplement Table 5. Ki67-index, immunohistochemistry

Day	**SO, mean ± SE**	**SE, mean ± SE**	***p*-value**
**0**	0.001±0.0002	0.002±0.0002	1.000
	**PH, mean ± SE**	**SH, mean ± SE**	***p*-value**
**1**	0.009±0.003	0.018±0.005	**<0.001***
**3**	0.08±0.01	0.1±0.02	0,2
**7**	0.04±0.008	0.071±0.01	**<0.001***
SO – sham splenectomy + 70% liver resection (n=6), SE - splenectomy + 70% liver resection (n=6), SH – splenectomy + hepatectomy (n=18), PH - sham splenectomy			

+ hepatectomy, *- statistically significant differences, p<0.05

Supplement Table 6. Flow cytometry, LSECs immunophenotype

CD31			
**Day**	**SO, mean ± SE, %**	**SE, mean ± SE,%**	***p*-value**
**0**	37.867±5.287	43.1±1.617	0.397
	**PH, mean ± SE, %**	**SH, mean ± SE, %**	***p*-value**
**1**	35.9±9.808	37.1 ±1.706	0.845
**3**	36.1±2.61	42.767±5.12	0.311
**7**	47.5±3.816	36.333±2.069	0.062
**VCAM-1 (CD106)**			
**Day**	**SO, mean ± SE, %**	**SE, mean ± SE,%**	***p*-value**
**0**	5.183±2.344	6.557±0.787	0.608
	**PH, mean ± SE, %**	**SH, mean ± SE, %**	***p*-value**
**1**	10.55±0.45	7.393±0.189	**0.005***
**3**	4.12±0.441	8.21±2.402	0.16
**7**	11.433±1.67	3.32±0.68	**0.035***
**CD49e**			
**Day**	**SO, mean ± SE, %**	**SE, mean ± SE,%**	***p*-value**
**0**	76.6±5.108	82.767±1.785	0.318
	**PH, mean ± SE, %**	**SH, mean ± SE, %**	***p*-value**
**1**	52.533±9.307	38.967±7.483	0.319
**3**	66.333±5.822	61.267±5.497	0.561
**7**	69.567±5.505	58.733±1.299	0.128
**VE-cadherin (CD144)**			
**Day**	**SO, mean ± SE, %**	**SE, mean ± SE,%**	***p*-value**
**0**	65.333±0.882	69.4±0.635	**0.02***
	**PH, mean ± SE, %**	**SH, mean ± SE, %**	***p*-value**
**1**	59.967±1.633	61.767±0.561	0.356
**3**	67.6±1.888	62.933±1.964	0.162
**7**	68.5±0.603	65.033±0.333	**0.007***
**Ki67**			
**Day**	**SO, mean ± SE, %**	**SE, mean ± SE,%**	***p*-value**
**0**	1.4±0.341	1.16±0.227	0.169
	**PH, mean ± SE, %**	**SH, mean ± SE, %**	***p*-value**
**1**	0.533±0.0949	1.287±0.174	**0.019***
**3**	0.727±0.402	2.22±0.2	**0.029***
**7**	0.620±0.244	0.900±0.211	0.435
**Annexin+PI**			
**Day**	**SO, mean ± SE, %**	**SE, mean ± SE,%**	***p*-value**

**0**	13.167±0.441	14.5±2.3	1.000
	**PH, mean ± SE, %**	**SH, mean ± SE, %**	***p*-value**
**1**	40,8±6,243	12,857±3,83	**0,019***
**3**	19,0±2,082	21,667±7,446	0,748
**7**	23,333±0,882	21,533±2,054	0,466
SO – sham splenectomy + 70% liver resection (n=6), SE - splenectomy + 70% liver resection (n=6), SH – splenectomy + hepatectomy (n=18), PH - sham splenectomy + hepatectomy, *- statistically significant differences, p<0,05			

Supplement Table 7. Flow cytometry, **CD3**+lymphocytes

**Day**	**SO, mean ± SE, %**	**SE, mean ± SE, %**	***p*-value**
**0**	0,305±0,09	0,233±0,0577	0,682
	**PH, mean ± SE, %**	**SH, mean ± SE, %**	***p*-value**
**1**	1.477±0.33	2.167±1	0.488
**3**	3.283±1.2	1.647±0.439	0.091
**7**	2.763±0.9	0.163±0.0289	**0.011***
SO – sham splenectomy + 70% liver resection (n=6), SE - splenectomy + 70% liver resection (n=6), SH – splenectomy + hepatectomy (n=18), PH - sham splenectomy + hepatectomy, *- statistically significant differences, p<0.05			

Supplement Table 8. Comparison of the absolute number of NK1.1 cells

**Group**	**Mean**	**Std Dev**	**P**
PH _1day	19.3	11.1	
SH _1day	11.3	7.5	0.3
PH _3day	**64.7**	12.2	
SH _3day	**21.7**	9.4	**0.009***
PH _7day	**26.3**	10.2	
SH _7day	**3.3**	1.1	**0.018***
SO_0day	18	20.8	
SE_0day	19	9.8	0.9

SO – sham splenectomy + 70% liver resection (n=6), SE - splenectomy + 70% liver resection (n=6), SH – splenectomy + hepatectomy (n=18), PH - sham splenectomy + hepatectomy, *- statistically significant differences, p<0.05

File Name count: 24	Labeling Controls Threshold	Hybridization Controls Threshold	Pos vs Neg AUC Threshold	Condition	polya spike-AFFX-r2-Bs...	polya spike-AFFX-r2-Bs...	polya spike-AFFX-r2-Bs...	polya spike-AFFX-r2-Bs-...	pos vs neg auc
1end_MUS_(Clariom_S_Mouse).sst...	Pass	Pass	Pass	3	6,52	7,79	8,57	9,63	0,78
2end_MUS_(Clariom_S_Mouse).sst...	Pass	Pass	Pass	3	6,58	8,02	8,72	9,97	0,8
3end_MUS_(Clariom_S_Mouse).sst...	Pass	Pass	Pass	3	6,18	7,26	8,04	9,3	0,83
4end_MUS_(Clariom_S_Mouse).sst...	Pass	Pass	Pass	4	6,49	7,5	8,18	9,15	0,85
5end_MUS_(Clariom_S_Mouse).sst...	Pass	Pass	Pass	4	6,05	7,44	8,12	9,23	0,85
6end_MUS_(Clariom_S_Mouse).sst...	Pass	Pass	Pass	4	6,44	7,68	8,22	9,59	0,82
7end_MUS_(Clariom_S_Mouse).sst...	Pass	Pass	Pass	6	6,27	7,7	8,02	9,41	0,84
8end_MUS_(Clariom_S_Mouse).sst...	Pass	Pass	Pass	6	5,96	6,84	7,38	8,64	0,84
9end_mus_(Clariom_S_Mouse).sst-...	Pass	Pass	Pass	6	5,87	7,16	7,69	8,94	0,84
10end_mus_(Clariom_S_Mouse).sst...	Pass	Pass	Pass	1	5,84	7,3	7,87	8,97	0,82
11end_mus_(Clariom_S_Mouse).sst...	Pass	Pass	Pass	1	6,09	7,76	8,02	9,44	0,8
12end_mus_(Clariom_S_Mouse).sst...	Pass	Pass	Pass	1	7,23	8,83	9,28	10,8	0,83
13end_mus_(Clariom_S_Mouse).sst...	Pass	Pass	Pass	2	6,43	7,89	8,27	9,7	0,81
14end_mus_(Clariom_S_Mouse).sst...	Pass	Pass	Pass	2	6,47	7,55	8,3	9,36	0,82
15end_mus_(Clariom_S_Mouse).sst...	Pass	Pass	Pass	2	6,19	7,66	8,07	9,41	0,83
16end_mus_(Clariom_S_Mouse).sst...	Pass	Pass	Pass	5	6,19	7,48	7,88	8,92	0,83
17end_MUS_(Clariom_S_Mouse).ss...	Pass	Pass	Pass	5	6,3	7,74	8,55	9,62	0,85
18end_MUS_(Clariom_S_Mouse).ss...	Pass	Pass	Pass	5	6,07	7,34	8,07	9,33	0,84
19end_MUS_(Clariom_S_Mouse).ss...	Pass	Pass	Pass	8	6,1	6,9	7,49	8,61	0,83
20end_MUS_(Clariom_S_Mouse).ss...	Pass	Pass	Pass	8	5,93	7,21	7,83	9,35	0,82
21end_MUS_(Clariom_S_Mouse).ss...	Pass	Pass	Pass	8	5,82	7,03	7,56	9,07	0,81
22end_MUS_(Clariom_S_Mouse).ss...	Pass	Pass	Pass	7	6,41	8,59	8,87	10,56	0,81
23end_MUS_(Clariom_S_Mouse).ss...	Pass	Pass	Pass	7	5,82	7,3	7,65	9,33	0,81
24end_MUS_(Clariom_S_Mouse).ss...	Pass	Pass	Pass	7	7,52	9,35	9,63	11,1	0,8

Supplement Figure7. Screenshot from the TAC software for microarray data analysis with quality control results.

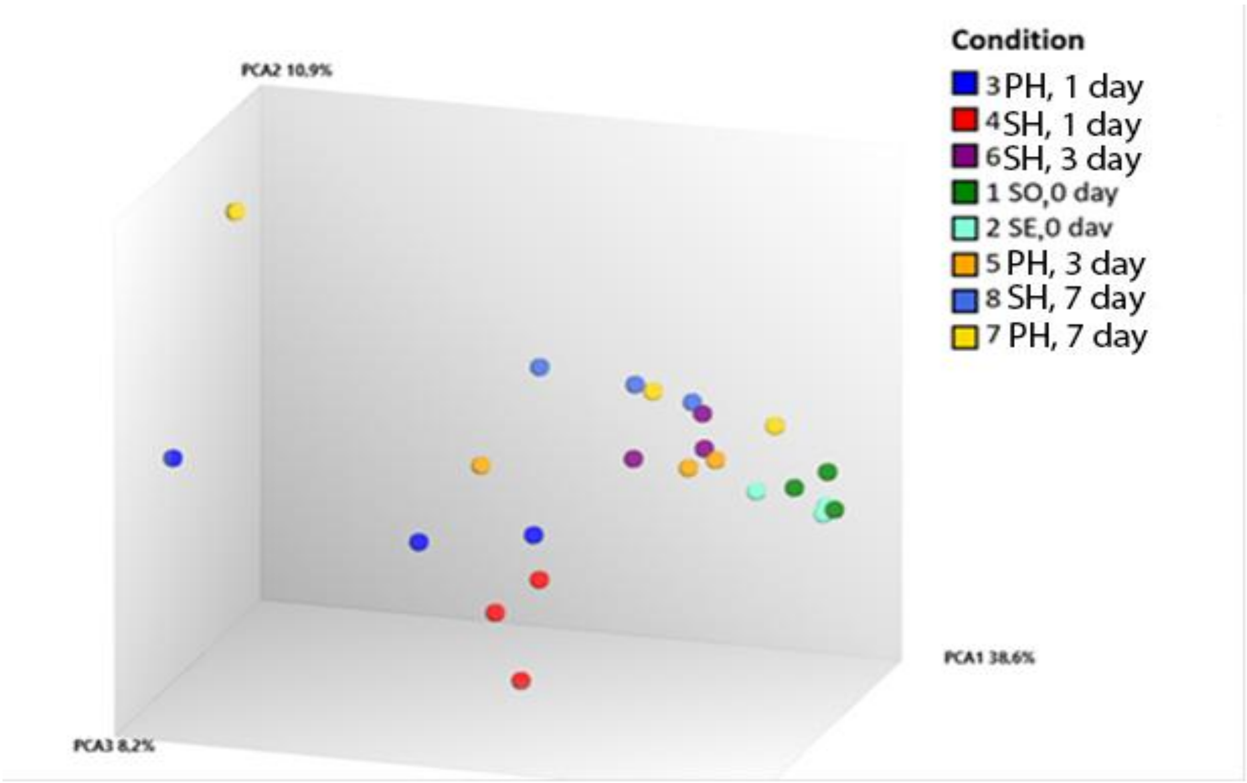

Supplement Figure 8 Principal component analysis (PCA) based on transcriptome data from microarray samples. SO – sham splenectomy + sham hepatectomy (n=3), SE - splenectomy, (n=3), SH – splenectomy + hepatectomy (n=9), PH - sham splenectomy + hepatectomy (n=9)

Supplement Table 9. LSECs relative gene expression

	il1b		
**Day**	**SO, mean ± SE**	**SE, mean ± SE**	***p*-value**
**0**	0.14±0.0658	0.232±0.0585	0.825
	**PH, mean ± SE**	**SH, mean ± SE**	***p*-value**
**1**	0,321±0,279	0,0547±0,0203	**0,003***
**3**	0,846±0,18	0,0157±0,00172	**<0,001***
**7**	0,538±0,0627	2,421±0,814	**0,002***
	il6		
**Day**	**SO, mean ± SE**	**SE, mean ± SE**	***p*-value**
**0**	0,0497±0,00961	0,0274±0,00201	**0,046***

	PH, mean ± SE	SH, mean ± SE	*p*-value
**1**	0.613±0.254	0.109±0.0182	0.052
**3**	0.0174±0.00144	0.00987±0.00161	**0.003***
**7**	0.0168±0.00325	0.137±0.0661	0.478
	Cx3cr1		
**Day**	**SO, mean ± SE**	**SE, mean ± SE**	***p*-value**
**0**	0.0206±0.00254	0.0137±0.00139	0.028
	**PH, mean ± SE**	**SH, mean ± SE**	***p*-value**
**1**	0.129±0.0399	0.00787±0.00197	**<0.001***
**3**	0.00915±0.00183	0.00238±0.000249	**<0.001***
**7**	0.0184±0.00479	0.063±0.0283	0.723
	Ccr2		
**Day**	**SO, mean ± SE**	**SE, mean ± SE**	***p*-value**
**0**	0.00275±0.00028	0.00973±0.00208	**<0.001***
	**PH, mean ± SE**	**SH, mean ± SE**	***p*-value**
**1**	0.111±0.0331	0.0019±0.00027	**<0.001***
**3**	0.00871±0.000657	0.0418±0.000171	0.269
**7**	0.00871±0.00028	0.0418±0.00208	0.289
	Ccl6		
**Day**	**SO, mean ± SE**	**SE, mean ± SE**	***p*-value**
**0**	0.379±0.064	0.367±0.0355	0.329
	**PH, mean ± SE**	**SH, mean ± SE**	***p*-value**
**1**	17.006±5.935	1.674±0.279	**<0.001***
**3**	0.27±0.0176	0.336±0.0403	0.868
**7**	0.263±0.0525	0.994±0.907	0.157
	Ccl9		
**Day**	**SO, mean ± SE**	**SE, mean ± SE**	***p*-value**
**0**	0.0249±0.00481	0.0325±0.0056	0.315
	**PH, mean ± SE**	**SH, mean ± SE**	***p*-value**
**1**	3.12±0.00751	0.235±0.00276	**<0.001***
**3**	0.081±0.00751	0.0188±0.00751	**<0.001***
**7**	0.0353±0.00841	0.165±0.0711	0.231
	Ccl4		
**Day**	**SO, mean ± SE**	**SE, mean ± SE**	***p*-value**
**0**	0.477±0.119	0.0983±0.00748	**<0.001***
	**PH, mean ± SE**	**SH, mean ± SE**	***p*-value**
**1**	1.928±0.514	0.495±0.0903	**0.002***
**3**	0.0959±0.0159	0.0517±0.00841	**0.026***
**7**	0.444±0.0897	0.144±0.0624	**0.014***
	Ccr1		
**Day**	**SO, mean ± SE**	**SE, mean ± SE**	***p*-value**

**0**	0.0157±0.00222	0.0582±0.00551	**<0.001***
	**PH, mean ± SE**	**SH, mean ± SE**	***p*-value**
**1**	2.183±1.128	0.144±0.0224	**0.027***
**3**	0.0668±0.0141	0.0395±0.00452	0.169
**7**	0.0893±0.00222	0.152±0.00551	0.353
*- statistically significant differences, *p*<0.05			
SO – sham splenectomy + 70% liver resection (n=6), SE - splenectomy + 70% liver resection (n=6), SH – splenectomy + hepatectomy (n=18), PH - sham splenectomy + hepatectomy, *- statistically significant differences, p<0.05			
